# Supplementary material for: Lung colonization by Aspergillus fumigatus is controlled by ZNF77
Source: Nat Commun. 2018 Sep 20;9:3835. doi: 10.1038/s41467-018-06148-7 (PMC6147781; doi:10.1038/s41467-018-06148-7)
Supplement: Supplementary file 1 — Supplementary Information [file 41467_2018_6148_MOESM1_ESM.pdf]

Supplementary Information

Aspergillus fumigatus colonization of lungs is controlled by ZNF77

Gago et al

Supplementary Note 1:

Initial Variant selection

To investigate genetic susceptibility factors to ABPA, DNA from 96 patients with ABPA and 167 asthmatic controls were whole exome sequencing as described in [1] and compared with the European controls in the 1000 genomes dataset. This discovery experiment, demonstrated a possible association of the genetic variant rs35699176 (Chr19:2936537, ZNF77) (Supplementary Table 1) with ABPA. Although we failed to replicate this variant using HiSeq, in a different population of 96 ABPA and 96 asthmatics, we found that it was present in ABPA patients carrying high fungal loads in their respiratory airways (30 % of patients (9/32) with an sputum Aspergillus positive PCR and 0% (0/13) patients with an *Aspergillus* negative PCR (p=0.036); and 60% (4/6 vs 0/15) of patients with fungal loads higher than 100 genome counts in bronchoalveolar lavage (p=0.0006). This variant was therefore associated with increased fungal burden rather than with ABPA per se (Tables 1 and 2, Figure 4). Functional analysis on this variant in the ZNF77 gene was performed as rs35699176 results in a premature stop codon which made it a good candidate for phenotypic characterization.

Supplementary Tables

Supplementary Table 1: Association analysis for the rs35699176 genetic variant between ABPA (n =97_, asthma (n= 167)and the 1000 genome dataset. FET: Fischer´s Exact Test P-value. AF: Allele Frequency

			Original population			Replication population		
Rs number (dbSNP)	Ref/Alt	Gene	Alt allele freq in ABPA	Alt allele freq in Asthma	FET	Alt allele freq in ABPA	Alt allele freq in Asthma	FET
rs35699176	G/A	ZNF77	9.3%	0.8%	1.52×10^{-5}	3.1%	4.7%	n/s

Supplementary Table2

Sequence of the CRISPR guides, repair template and primers used in this study. RT: Repair template, Rs35699176-1 and Rs35699176-2 CRIPSR guides.

Name	Aim	Sequence 5-3´	Reference
Rs35699176-1	CRISPR guide	TCAGATGCCTCTGTGGGATTGTTTT	This work
Rs35699176-1	CRISPR guide	AATCCCACAGAGGGCATCTGACGGTG	This work
RT_rs35699176	Repair template	CGGAGAGGCCCATCCCTCCGGTTGAGCGCCACTCACCTCAGATGCCTCTATG GGATTGATGCTGATCTCCAGTGTTATCAAATCTCCAATTTTCTCCAA	This work
Rs35699176-3	ZNF77 genotyping	TCTCTGTGGGTCTGAAATTGTGT	This work
Rs35699176-4	ZNF77 genotyping	TGAAAAGAAATCCGTATGTGGTCT	This work
ZNF77_2934825	ZNF77 expression	CAGCTGACTTCTCACCTCAG	This work
ZNF77_2936594	ZNF77 expression	CACAGGAAGTGATTCCTGGT	This work
GAPDH_F	Gene expression	GTCTTCACCACCATGGAGA	[2]
GAPDH_R	Gene expression	CCAAAGTTGTCATGGATGACC	[2]
CAV	Gene expression	N/A	Biorad
ECAD	Gene expression	N/A	Biorad
OCLUD	Gene expression	N/A	Biorad

Supplementary Table 3

Fungal strains used in this work.

Species	Strain	Origin
Aspergillus fumigatus	CEA10	CBS144.89 (Isolated by JP Latge)
Aspergillus fumigatus	A1160	CEA10 DeltaKU80 pyrG-
Aspergillus fumigatus	Af293	University Hospital of South Manchester
Aspergillus fumigatus	3MD	Strain 9.20152 National Aspergillosis Center, Manchester UK.
Aspergillus fumigatus	Δ Asp f 13	Michael Bromley gift
Aspergillus fumigatus	Δ Asp f 5	Michael Bromley gift
Aspergillus nidulans	KE12	Michael Bromley gift
Aspergillus terreus	MRCM67020	University Hospital of South Manchester
Aspergillus niger	MRCM44001	University Hospital of South Manchester

Supplementary Figures:

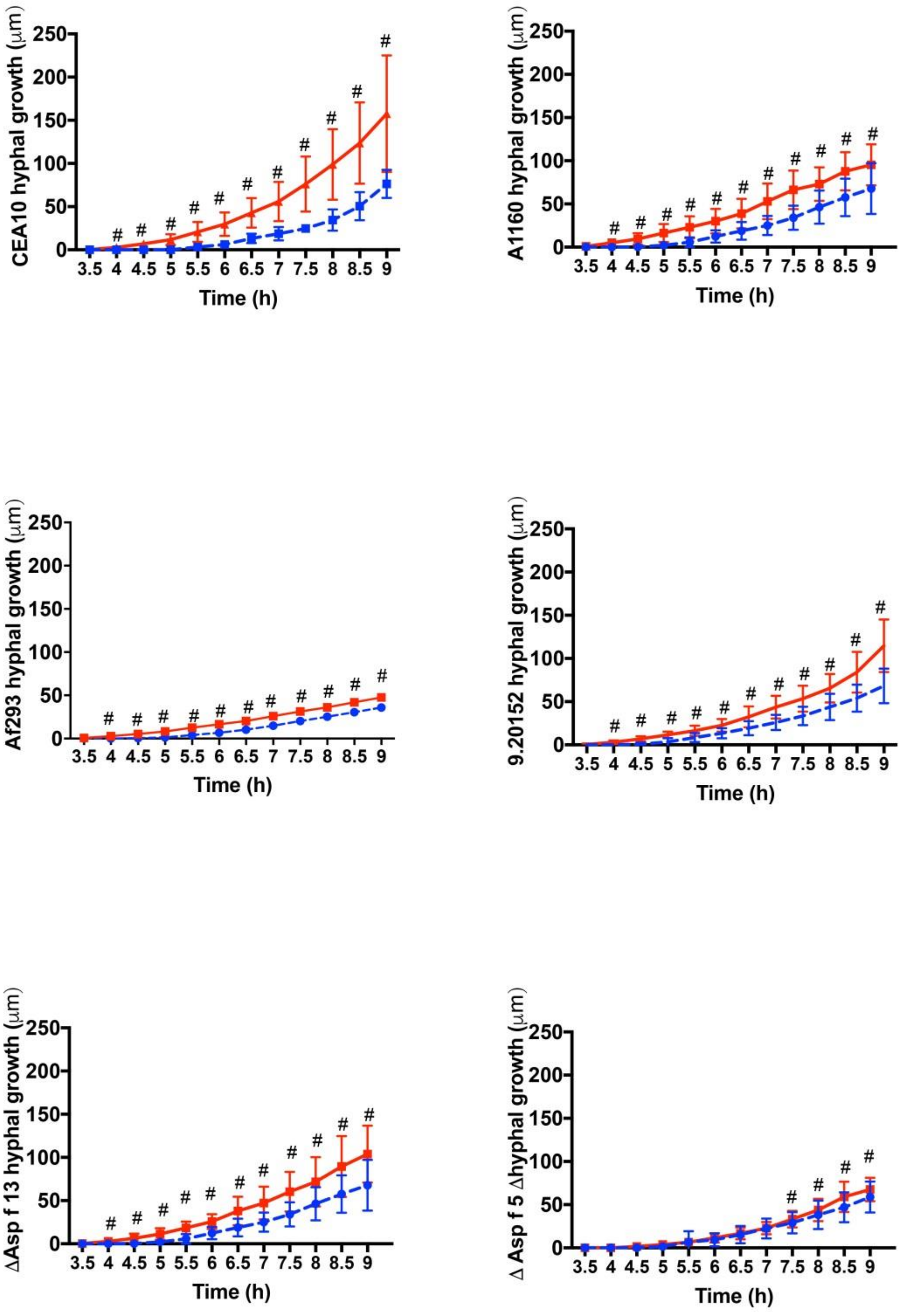

Supplementary Figure 1: *A. fumigatus* hyphal extension during infection 16HBE and 16HBE$^{\text{rs35699176 of}}$ bronchial epithelial cells; Multiple comparison t test were used to detect differences in growth for each time point represented as mean and standard deviation # indicates P <0.05.

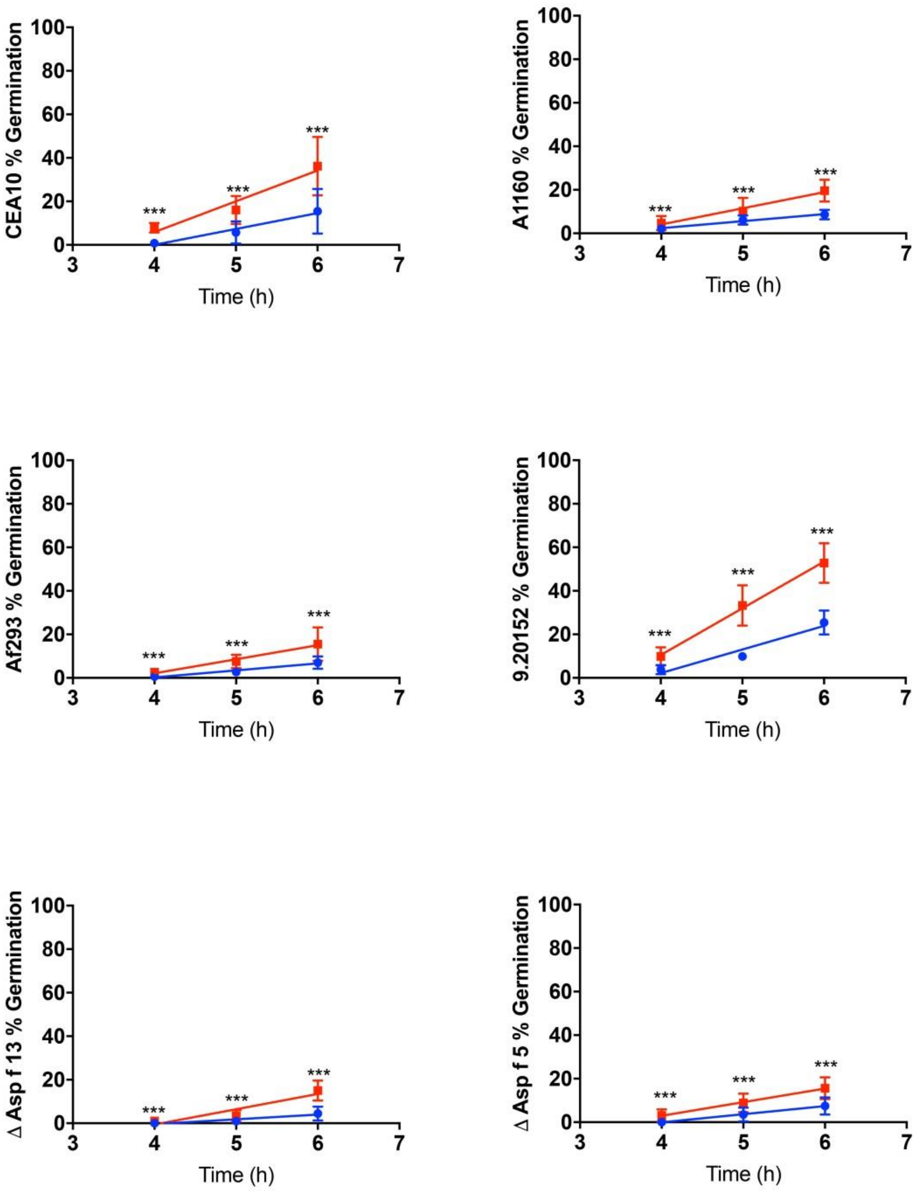

Supplementary Figure 2: Percentage *A. fumigatus* germination during infection of bronchial epithelial cells.

A. fumigatus germination time points (h) determined by time-lapse microscopy. Mean ± SD is represented. Linear regression analysis and the Pearson correlation test were performed to compare germination time between cell lines. Data points represent mean and standard deviation; *** indicates P <0.001.

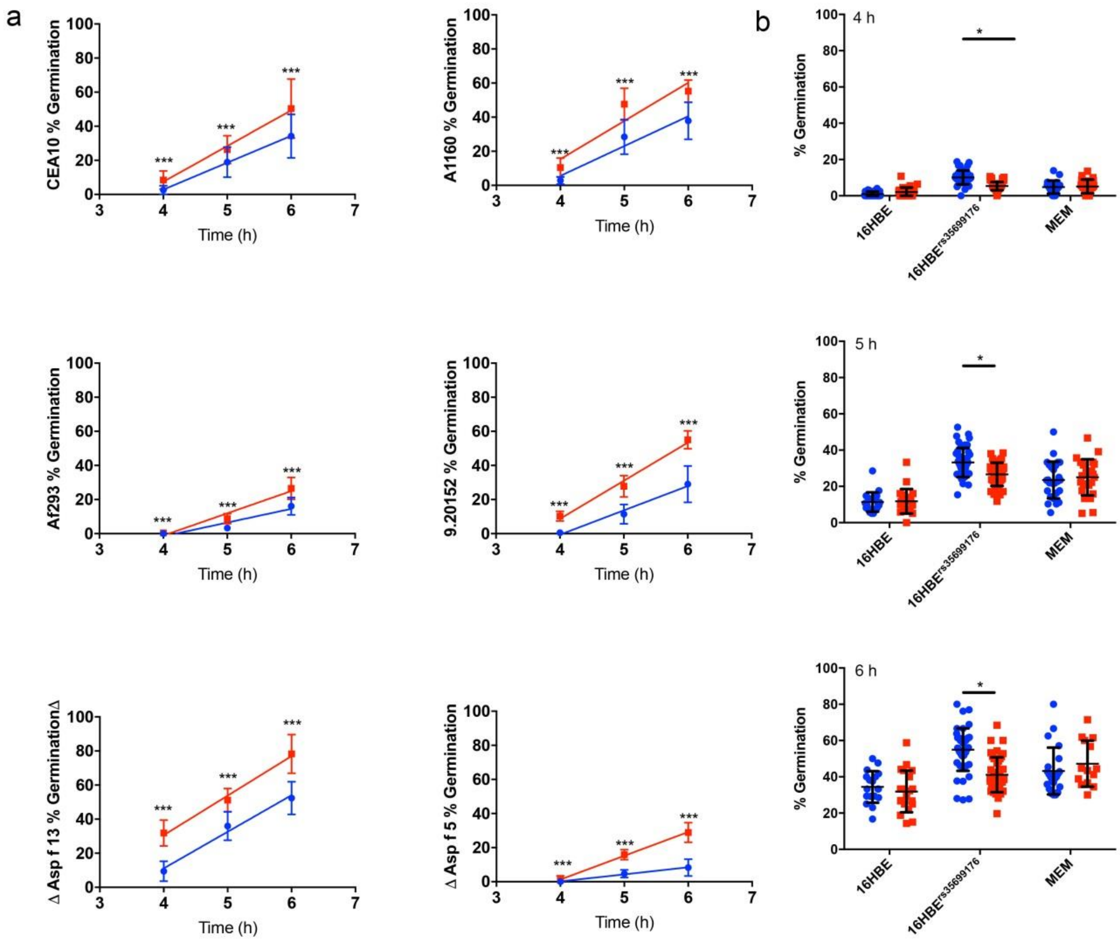

Supplementary Figure 3: *A. fumigatus* challenge assays in cell culture supernatants. Data points represent mean and standard deviation.

A. *A. fumigatus* germination time points (h) in cell culture supernatants from 16HBE and 16HBE^{rs35699176}. Mean ± SD is represented. Linear regression analysis and the Pearson correlation test were performed to compare germination time between cell lines; *** indicates P <0.001. Blue: 16HBE, Red 16HBE^{rs35699176}

B. Heat treatment delays *A. fumigatus* CEA10 germination time in 16HBE^{rs35699176} cell lines. Results are expressed as mean ± SD of three experiments assayed in triplicate. Comparisons were performed using two way anova with multiple comparison test. Statistically significant differences: * P < 0.05. Blue: native conditions, Red: heat treatment

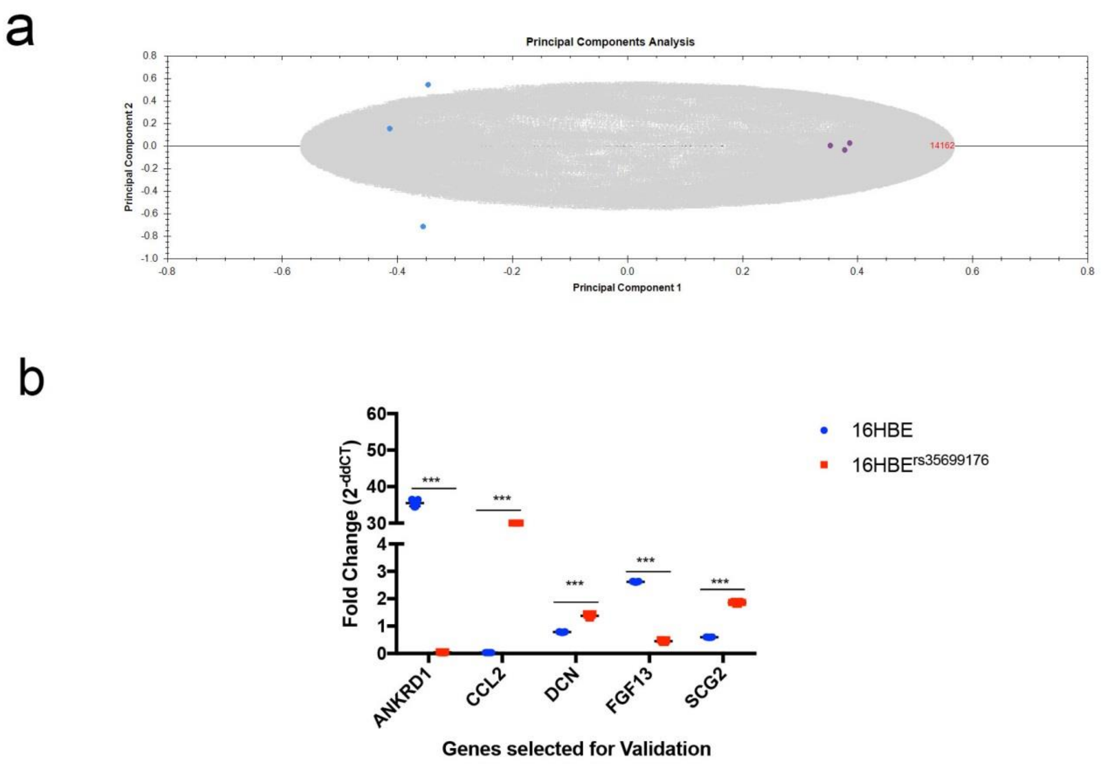

	Discovery?	P value	Mean1	Mean2	Difference	SE of difference	t ratio	df	q value
ANKRD1	Yes	<0.000000000000001	35.48	0.04334	35.44	0.2887	122.7	16	<0.000000000000001
CCL2	Yes	<0.000000000000001	0.03541	29.55	-29.52	0.1304	226.3	16	<0.000000000000001
DCN	Yes	0.000000000000004	0.7815	1.378	-0.5965	0.02099	28.41	16	0.000000000000004
FGF13	Yes	<0.000000000000001	2.619	0.4461	2.173	0.01571	138.3	16	<0.000000000000001
SCG2	Yes	<0.000000000000001	0.5939	1.856	-1.262	0.01292	97.7	15	<0.000000000000001

Supplementary Figure 4: Principal component analysis and RNA-seq validation

(A) Principal component analysis matrix for 16HBE and 16HBE^{rs35699176} indicates no differences within but a significant difference between groups. Three biological replicates were assessed: 16HBE (purple) and 16HBE^{rs35699176} (blue) cells.

(B) RNA-seq validation. Five genes were selected for replication based on p-adjusted value and function: Ankyrin Repeat Domain 1 (ANKRD1), C-C Motif Chemokine Ligand 2 (CCL2), decorin (DCN), fibroblast growing factor (FGF3) and secretogranin (SGG2). Two way ANOVA with multiple comparison test were used for comparison; Data represents mean and standard deviation *** = $p < 0.0001$. Normalization was done using GAPDH as a housekeeping gene. Blue: 16HBE, Red 16HBE^{rs35699176}

Supplementary References:

1. Overton NLD, Brakhage AA, Thywißen A, Denning DW, Bowyer P. Mutations in EEA1 are associated with allergic bronchopulmonary aspergillosis and affect phagocytosis of Aspergillus fumigatus by human macrophages. *PLoS ONE* 2018 **13**(3): e0185706.

2. O'Driscoll KE, Hatton W, Burkin HR, Leblanc N, Britton F. Expression, localization, and functional properties of Bestrophin 3 channel isolated from mouse heart. Am J Physiol Call Physiol 2008, **295** (6): C1610-C1624
