# Supplementary material for: Lung colonization by Aspergillus fumigatus is controlled by ZNF77
Source: Nat Commun. 2018 Sep 20;9:3835. doi: 10.1038/s41467-018-06148-7 (PMC6147781; doi:10.1038/s41467-018-06148-7)
Supplement: Supplementary file 3 — Description of Additional Supplementary Files [file 41467_2018_6148_MOESM3_ESM.pdf]

Description of Additional Supplementary Files

File Name: Supplementary Movie 1
Description: 3D view of 16HBE bronchial epithelial cells section by using confocal microscopy. Cell monolayers are confluent and adhered to the plate.

File Name: Supplementary Movie 2:
Description: 3D view of 16HBErs35699176 bronchial epithelial cells section by using confocal microscopy. Cell monoloayers are not confluent and cannot adhere to the substrate.

File Name: Supplementary Movie 3
Description: 16HBE bronchial epithelial cell lines co-cultured with A. fumigatus CEA10 at 0.1 MOI. Imaging was recorded using confocal microscopy. Notice that A. fumigatus germination becomes noticeable at 5.5 h after infection.

File Name: Supplementary Movie 4
Description: 16HBErs35699176 bronchial epithelial cell lines co-cultured with A. fumigatus CEA10 at 0.1 MOI. Imaging was recorded using confocal microscopy. Notice that A. fumigatus germination becomes noticeable at 4.5 h after infection.

File Name: Supplementary Data 1:
Description: Secretome analysis of 16HBE and 16HBErs35699176

File Name: Supplementary Data 2
Description: Pairwise comparison of RNA-seq data of 16HBE and 16HBErs35699176

File Name: Supplementary Data 3
Description: Pairwise comparison of RNA-seq data of 16HBE and 16HBE infected with Aspergillus fumigatus

File Name: Supplementary Data 4
Description: Pairwise comparison of RNA-seq data of 16HBE and 16HBErs35699176 infected with Aspergillus fumigatus

File Name: Supplementary Data 5
Description: Pairwise comparison of RNA-seq data of 16HBErs35699176 and 16HBErs35699176 infected with Aspergillus fumigatus
